# *Transplantation Direct*: The Official Journal of the International Society of Vascularized Composite Allotransplantation

**DOI:** 10.1097/TXD.0000000000001902

**Published:** 2026-03-19

**Authors:** Jérôme Duisit, Edward Geissler, Patrik Lassus, Hatem Amer

**Affiliations:** 1 Deputy Editor of Transplantation Direct, VCA Section.; 2 International Society of Vascularized Composite Allotransplantation (ISVCA), Montreal, QC, Canada.; 3 Department of Plastic and Reconstructive Surgery, Iris South Hospitals, Brussels, Belgium.; 4 Editor of Transplantation Direct.; 5 Executive Editor of Transplantation.; 6 Department of Surgery, University Hospital Regensburg, University of Regensburg, Regensburg, Germany.; 7 Department of Plastic Surgery, University of Helsinki and Helsinki University Hospital, Helsinki, Finland.; 8 Division of Nephrology and Hypertension, Department of Medicine, Mayo Clinic, Rochester, MN.; 9 The William J von Liebig Center for Transplantation and Clinical Regeneration.

In December 2024, the council of the International Society for Vascularized Composite Allotransplantation (ISVCA), a section of the Transplantation Society (TTS), designated *Transplantation Direct* as the official journal of the Society. The journal was launched in 2015 as the sister open-access journal of *Transplantation*, with the ancillary objective of hosting TTS sections.

*Transplantation Direct* represents the optimal platform for ISVCA due to its strong relationship with TTS and its partnership with the journal *Transplantation,* which features an effective quality peer-review process.^1^ It addresses both the scientific integrity and reaches the community that ISVCA envisions. The partnership between ISVCA and *Transplantation Direct* is an important opportunity to strengthen communication in VCA with the wider transplant community, fostering talent, research, and broader-scale activities in the field of VCA.

Designating *Transplantation Direct* as the official journal of ISVCA required a deputy editor for VCA. A call was made to the membership of ISVCA, and Dr Jérôme Duisit was selected.

The VCA Deputy Editor’s duties are as follow:

- Promote investigators from the membership of ISVCA to publish articles in *Transplantation Direct*.
- Share knowledge gained during official society congresses, including meeting reports, consensus guidelines, or recommendations that arise from scientific roundtables at the meetings, and identify topics that should be submitted as regular articles for publication.
- Join in assessing articles on VCA topics in the journal *Transplantation* being considered for transfer to *Transplantation Direct*.
- Be the handling section editor for all transferred and direct submissions on the topic of VCA in *Transplantation Direct*.

To adequately address the complexity of the field of VCA, *Transplantation Direct* offers the advantage of broad coverage on interdisciplinary topics in transplantation, allowing ISVCA membership to tap into the various areas of the field. The area of VCA has characteristics that differ considerably from the traditional solid organ transplantation field. As the transplanted “organ” is not homogeneous and can vary substantially in morphology and function, such as face, limbs, genitalia, larynx, whole eye, or the abdominal wall. Most of these organs are visible and are life-enhancing rather than lifesaving. However, VCA and solid organ transplantation have much in common, such as alloreactivity with the need for immunosuppression and its complications. There is also the need to procure organs from deceased donors.

In the TTS journal family, we hope to especially promote several VCA themes:

- Psychosocial, quality of life, and ethical questions specific to VCA.
- Donation and procurement of vascularized composite transplants.
- Machine perfusion and other modalities supporting organ preservation.
- Surgical techniques, virtual surgical planning, and execution.
- Improving function with physical and occupational therapy.
- Immunological aspects and immunosuppression strategies in VCA.
- Methods of assessing patient outcomes.
- Tissue engineering approaches in composite tissue reconstruction, such as 3-dimensional bioprinting and decellularization/recellularization techniques.

To better represent what we hope to publish in the TTS family of journals, our subject designation has been modified from “Hand and Composite Tissue Allotransplantation” to “Vascularized Composite Allotransplantation and Engineering.” This change broadens our aim to cover more aspects of clinical and experimental progress in the field.

Please stay tuned to the *Transplantation Direct* website for the most noteworthy information and research related to ISVCA’s mission. Importantly, a collection of the most recent articles in the TTS journals is archived and directly accessible on the opening page of the *Transplantation Direct* website. We also welcome you to visit our ISVCA website at www.tts.org/isvca for more detailed information about the activities of our Society.

## References

[1] Geissler EK Chapman JR. *Transplantation Direct*: TTS ejournal progress update. Transplantation. 2020;104:1321. 32282659 10.1097/TP.0000000000003271

